# Benefit of a multimodal approach combining chemotherapy and surgery in oligometastatic gastric cancer: experience from a tertiary referral center

**DOI:** 10.3389/fonc.2024.1343596

**Published:** 2024-06-07

**Authors:** Maria Grazia Maratta, Antonio Vitale, Michele Basso, Raffaella Vivolo, Elena Di Monte, Alberto Biondi, Andrea Di Giorgio, Fausto Rosa, Vincenzo Tondolo, Annamaria Agnes, Giampaolo Tortora, Antonia Strippoli, Carmelo Pozzo

**Affiliations:** ^1^ Comprehensive Cancer Center, Fondazione Policlinico Universitario Agostino Gemelli IRCCS, Università Cattolica del Sacro Cuore, Rome, Italy; ^2^ Department of Surgery, Fondazione Policlinico Universitario Agostino Gemelli IRCCS, Università Cattolica del Sacro Cuore, Rome, Italy

**Keywords:** gastric cancer, induction chemotherapy, metastasectomy, surgical oncology, cancer survival

## Abstract

**Introduction:**

Gastric cancer (GC) is the fourth leading cause of cancer-related death worldwide with limited therapeutic options. The aim of this study was to analyze the value of adding surgery to the first-line treatment in patients with oligometastatic GC (OGC).

**Methods:**

This retrospective study included patients with OGC who underwent induction chemotherapy followed by surgery of both primary tumor and synchronous metastasis between April 2012 and April 2022. Endpoints were overall survival (OS) and relapse-free survival (RFS) analyzed by the Kaplan–Meier method. Prognostic factors were assessed with the Cox model.

**Results:**

Data from 39 patients were collected. All cases were referred to our multidisciplinary tumor board (MTB) to evaluate the feasibility of radical surgery. After a median follow-up of 33.6 months (mo.), median OS was 26.6 mo. (95% CI 23.8–29.4) and median RFS was 10.6 mo. (95% CI 6.3–14.8). Pathologic response according to the Mandard criteria (TRG 1–3, not reached versus 20.5 mo. for TRG 4–5; HR 0.23, p=0.019), PS ECOG ≤ 1 (26.7 mo. for PS ≤ 1 versus 11.2 mo. for PS >1; HR 0.3, p=0.022) and a low metastatic burden (26.7 mo. for single site versus 12.9 mo. for ≥2 sites; HR 0.34, p=0.039) were related to good prognosis. No major intraoperative complications nor surgery-related deaths occurred in our series.

**Discussion:**

A sequential strategy of preoperative chemotherapy and radical surgical excision of both primary tumor and metastases was demonstrated to significantly improve OS and RFS. Multidisciplinary evaluation is mandatory to identify patients who could benefit from this strategy.

## 1 Introduction

Gastric cancer (GC) represents the fifth most diagnosed cancer and the fourth leading cause of cancer-related death (1–3). For the treatment of advanced GC, current guidelines recommend a systemic chemotherapy strategy (4, 5) based on platinum and fluoropyrimidine triplets or doublets combined with anti-HER2 antibodies or immunotherapy based on the molecular assessment (6, 7). Despite these treatments, the median OS for metastatic disease is merely 13.8 months and less than 10% of the patients survive longer than 2 years (8, 9). Thus, new strategies are needed. The role of surgery in metastatic GC is an open debate. In patients identified as having oligometastatic GC (OGC), surgery with curative intention could provide benefit, particularly in those patients with an excellent response to preoperative chemotherapy, achieving the possibility of an R0 intervention (10–14). All available data come from retrospective studies and are controversial. Evidence from randomized controlled prospective trials, such as the REINASSANCE/FLOT-AIO 5 phase 3 study, is anticipated (NCT02578368) (15). The aim of our single-institution experience was to analyze the benefit of adding surgery as another therapeutic option in selected oligometastatic patients after systemic preoperative chemotherapy, retrospectively collecting data from patients treated in a high-volume center for GC treatment.

## 2 Materials and methods

### 2.1 Study design and population

This study applied the Reporting of studies Conducted using Observational Routinely-collected health Data (RECORD) statement (16). We retrospectively collected data from patients who underwent surgery for OGC in Fondazione Policlinico Universitario “Agostino Gemelli”—IRCCS, Rome, between April 2012 and April 2022. All patients were aged 18 years or older, with a new diagnosis of advanced {stage IV according to American Joint Committee on Cancer (AJCC) 8th edition (17)} gastric or gastroesophageal junction adenocarcinoma, an Eastern Cooperative Oncology Group (ECOG) performance status (PS) ≤ 2, and adequate baseline bone marrow, liver, and renal function. Tumors located proximally in the gastroesophageal junction were classified according to Siewert and Stein (18). Only Siewert type III tumors were included in the analysis. No one had received any previous chemotherapy for advanced disease and was considered eligible to start a doublet or triplet chemotherapy combination as a first-line treatment. All the radiological images and reports were revised at least twice, at diagnosis and preoperatively after induction chemotherapy by the multidisciplinary tumor board (MTB) to assess the TNM stage and disease response to medical treatment. The surgical indication for each OGC patient was discussed by our GC dedicated MTB and defined by the following criteria: (1) confirmed primary GC, (2) resectable hepatic metastasis, (3) minimal peritoneal dissemination [i.e., peritoneal cancer index (PCI) <6, uni- or bilateral Krukenberg tumors], (4) up to three lymph nodes involved in stations categorized as M1 according to AJCC manual 8th edition (17), (5) eligible for radical surgical treatment, and (6) in good general health conditions. Diagnostic laparoscopy and peritoneal cytology were performed to assess the presence of peritoneal dissemination in newly diagnosed advanced GC patients with suspected peritoneal dissemination before starting any kind of treatment as per routine clinical practice and PCI index was used to stratify patients according to living guidelines (4, 19). The decision about the chemotherapy administration was made by medical oncologists according to baseline disease and patient characteristics. The heterogeneity in treatment protocols and number of cycles performed were not considered for statistical analysis. Primitive tumor excision was combined with metastasectomy in one-time surgery. All patients with potentially curable lesions were treated by metastasectomy, gastrectomy, and extended lymphadenectomy (D2) on purpose, in agreement with current guidelines (4, 20). For tumors located in the middle and lower thirds of the stomach, a subtotal gastrectomy was generally preferred, provided that an adequate resection margin was maintained. After total gastrectomy with lymph node dissection, esophagojejunostomy (using a circular stapler, diameter 25 mm) was used routinely for Roux-en-Y reconstruction. In case of subtotal gastrectomy, intestinal continuity was restored by means of Billroth II or Roux-en-Y gastrojejunostomy, at the discretion of the surgeon. Resection was stated as potentially curative (R0 according to the UICC/AJCC staging system (17), if macro- and microscopically no tumor was left following surgery). At the end of the operation, the surgeon resected all lymph nodes from the surgical specimen and identified their distribution according to the Japanese Gastric Cancer Association classification (20). Pathological TNM classification, the status of margins and histological tumor regression grade (TRG) assessed in accordance with the Mandard criteria (21) were described in the pathologists’ report. The patients were monitored for up to 30 days by a surgeon to assess postoperative complications and mortality. All clinical and pathological data were stored in a GC database and retrospectively evaluated for this study. Patient status was investigated by follow-up examination or by telephone contact.

### 2.2 Endpoints

The primary endpoint was overall survival (OS) defined as the time from diagnosis to death or latest follow-up; the secondary endpoint was recurrence-free survival (RFS) defined as the time from surgery to radiological evidence of relapse. An exploratory analysis was performed to evaluate the survival impact of demographic and clinicopathological factors such as age, sex, ECOG PS, histotype (according to Lauren’s classification) (22, 23), primitive tumor site (esophagogastric junction, fundus/body, and antrum/pylorus) and extension (T), metastatic sites (liver, lymph nodes, and peritoneum) and burden (single metastatic site versus multiple sites), resection margins (R0 versus R1–2), and TRG according to the Mandard criteria (1–3 versus 4–5) (21, 24). Furthermore, we tested a prognostic score based on five relevant prognostic factors: (1) pathological tumor growth (T+) on surgical specimen; (2) presence of liver metastases (L+); (3) peritoneum (P+) or (4) non-locoregional lymph nodes (N+); and (5) presence of macroscopical residual disease on resection margin (R2 resection).

### 2.3 Statistical analysis

Statistical analyses were performed using SPSS^®^ software, version 29.0 Chicago, IL. The survival curves were generated by the Kaplan–Meier method and compared using the Log-rank test. Demographic and clinicopathological factors were collected and evaluated by univariate analysis among patients who received macroscopically complete resection to evaluate their impact on OS and RFS. Discrete variables were compared using the Chi-square test and continuous variables were compared using independent-samples *t*-test. *p*-value <0.05 was considered statistically significant. Only variables that were statistically significant at univariate analysis were tested in multivariate analysis with Cox proportional hazards model to identify independent predictors of specific survival and recurrence.

## 3 Results

### 3.1 Demographic and clinicopathological characteristics of patients

In the past 10 years, 39 patients with metastatic GC, 16 men and 23 women, matching the aforementioned criteria had undergone surgery with radical intent at our institution. Patients’ age at diagnosis ranged from 26 to 75 years, with a median age of 58 years. PS ECOG was 0–1 for 33 patients and 2 for 6 patients. Primary tumor site was located at the gastroesophageal junction in 8 cases, at the gastric body in 17 cases, and at the antrum/pylorus in 14 cases. All subjects had a histopathologically confirmed diagnosis of adenocarcinoma according to Lauren’s classification (22). The specimens of 14 patients were diagnosed as intestinal subtype, 15 were diffuse, 6 were mixed type, and 4 were indeterminate type. Signet ring cells have been detected in 13, all in patients with a diffuse subtype. Among the population, 18% of patients were found positive for HER2 amplification score ≥ 2+. All patients had a baseline clinical T4 stage except one T3, and most of them had a locoregional nodal involvement (N1–N2). A total of 34 study patients had only one metastatic site: 6 of them presented with only liver metastases, 22 presented with peritoneal metastases, and 6 presented with distant lymph nodes (**Table 1**). The others had ≥2 synchronous metastatic sites (**Table 2**). All of them were considered eligible to first-line chemotherapy and received 2–6 months of induction systemic platinum and fluoropyrimidine combination chemotherapy, with or without trastuzumab, according to histology and HER2 status (4, 5) (**Table 3**). Any case was referred to our MTB and considered amenable to radical surgery after the induction chemotherapy.

**Table 1:** Patients’ characteristics.

Characteristics	Patients, number (%)
Gender	
Male	16 (41)
Female	23 (59)
PS (ECOG)	
0–1	33 (85)
2	6 (15)
Primitive tumor location	
Gastroesophageal junction	8 (20)
Body/Fundus	17 (44)
Antrum/Pylorus	14 (36)
Histotype according to Lauren’s classification	
Intestinal	14 (36)
Diffuse	15 (38)
Mixed	6 (15)
Indeterminate	4 (11)
Signet ring cell presence	
No	26 (67)
Yes	13 (33)
HER-2 status	
0	28 (72)
1+	4 (10)
2+	1 (3)
3+	6 (15)
cT	
3	1 (2.6)
4	38 (97.4)
cN	
N0	5 (13)
N1	32 (82)
N2	2 (5)

**Table 2:** Metastatic site number and distribution.

Metastatic site number and distribution	Patients, number (%)
Single site	34 (87)
Liver	6 (17.5)
Peritoneum	22 (65)
Distant lymph nodes station	6 (17.5
Multiple sites	5 (13)
Liver + Peritoneum	1 (20)
Distant lymph node station + Peritoneum	4 (80)

**Table 3:** Peri-operative treatments. (A) Chemotherapy; (B) Radiotherapy. RT, radiotherapy.

Chemotherapy regimen	Patients, number (%)
Induction phase	
FLOT	17 (43.5)
FOLFOX	13 (33.5)
EOX	1 (2)
ECF	2 (5)
Cisplatin + fluorouracil	6 (15)
Trastuzumab	4 (10)
Adjuvant phase	
None	13 (33)
FLOT	7 (18)
FOLFOX	7 (18)
XELOX	1 (2.5)
FOLFIRI	1 (2.5)
Cisplatin + fluorouracil	1 (2.5)
Capecitabine	1 (2.5)
DeGramont	1 (2.5)
Trastuzumab	3 (8)
(**a**)	
Radiotherapy	Patients, number (%)
RT alone	1 (2.5)
Concomitant RT with capecitabine	2 (5)
Sequential RT (after adjuvant chemotherapy	3 (8)
(**b**)	

### 3.2 Surgical outcomes

Surgery was conducted in both primitive tumor and metastases at the same time. Regarding surgical approach to primitive tumor, for 59% of patients, a total gastrectomy was performed, while in 41%, a subtotal gastrectomy was carried out. Four (10.2%) patients with GEJ adenocarcinoma underwent a thoraco-abdominal gastrectomy, while in all other cases, a laparoscopic abdominal surgical approach was used. In 28 (71.8%) cases, a laparoscopic technique was preferred, whereas in 11 (28.2%) cases, patients underwent a laparotomy. Up to 87% of cases underwent a lymphadenectomy ≥ D2; in five cases, a D1 lymphadenectomy was performed. A total of 19 patients (49%) presenting peritoneal-only metastatic spread underwent hyperthermic intraperitoneal chemotherapy (HIPEC). HIPEC was carried out according to the Coliseum technique (25) using mitomycin C (MMC) at a dose of 15 mg/m^2^ and cisplatin at a dose of 75 mg/m^2^ administered for 90 min with an inflow temperature of 41–42°C and an outflow temperature of 39–40°C (26). An R0 resection was achieved in 27 patients (69%), while microscopic (R1) or macroscopic (R2) residual disease was found in 7 (18%) and 5 (13%) cases, respectively (**Table 4**). TRG evaluated according to the Mandard criteria was available for 26 patients: 58% of them were pathologic responders (TRG 1–3) and 42% were non-responders (TRG 4–5). Combined surgery on both gastric primitive tumor and metastasis clearly affected operation duration but did not negatively affect patients’ outcome in terms of mortality and performance status. No major intraoperative complications or surgery-related deaths occurred in our series. During the hospitalization, up to 12 (30.7%) patients developed at least one postoperative complication. According to the Clavien–Dindo classification (27), the most frequent were grade I/II complications, while two patients (5.1%) reported a grade III complication. One patient reported an abdominal collection treated by percutaneous drainage plus antibiotic therapy; another experienced postoperative bleeding treated by angioembolization. Only one subject (2.5%) reported a grade IV complication: an acute renal failure requiring hemodialysis. A summary of postoperative complications is available in the **Supplementary Materials** (**Supplementary Table S1**). Actually, the greater surgical stress related to an extensive resection did not deteriorate prognosis in patients without postoperative complications (69.2%). After surgery, 26 patients (66.7%) were able to receive an adjuvant treatment (**Table 3**).

**Table 4:** Surgical outcomes.

Surgical outcomes	Patients, number (%)
Gastrectomy	
Total	23 (59)
Subtotal	16 (41)
Lymphadenectomy	
D1	5 (13)
D2	29 (74)
D3	5 (13)
pT	
0	2 (5)
1	1 (3)
2	3 (8)
3	11 (28)
4	22 (36)
pN	
0	8 (20.5)
1	8 (20.5)
2	8 (20.5)
3	15 (38.5)
Residual disease	
R0	27 (69)
R1	7 (18)
R2	5 (13)
Tumor regression grade (Mandard)	
1	3 (8)
2	0 (0)
3	12 (31)
4	8 (20)
5	3 (8)
Undefined on pathology report	13 (33)
HIPEC	
Yes	19 (49)
No	20 (51)
Recurrence rate	
Yes	28 (72)
No	11 (28)
Site of recurrence	
Peritoneum	22 (78)
Liver	3 (11)
Other	3 (11)

pT, pathological primary tumor classification; pN, pathological lymph node involvement classification.

### 3.3 Survival outcomes

**Figure 1:**
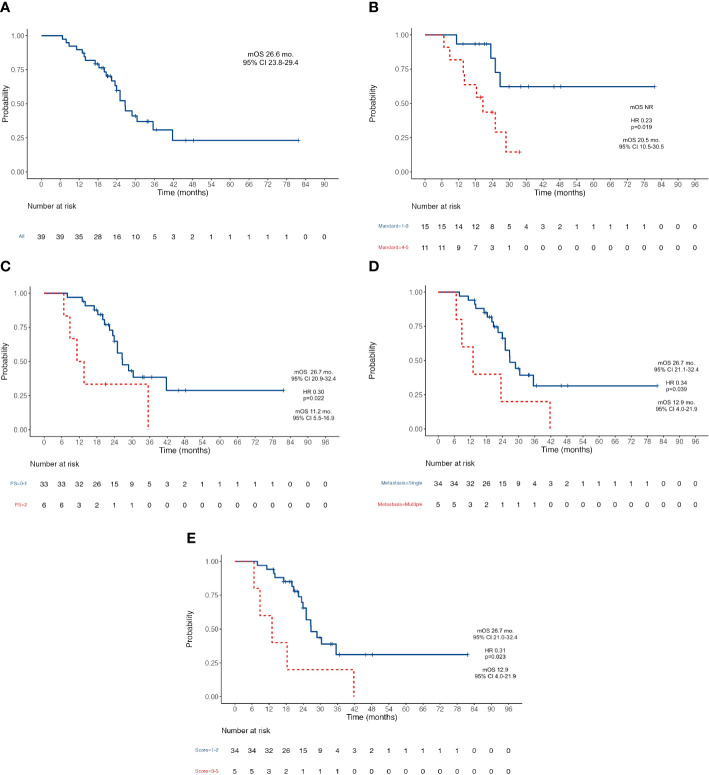
Kaplan-Meier survival curves for OS. **(A)** OS in the whole population; **(B)** OS according to Mandard Tumor Regression Grade after induction chemotherapy; **(C)** OS according to patient’s PS ECOG; **(D)** OS according to the number of metastatic sites; **(E)** OS according to the five-factors prognostic score. OS, Overall Survival; NR, Not Reached; PS, Performance Status; ECOG Eastern Cooperative Oncology Group; mOS, Median Overall Survival; mo, Months; CI, Confidence Interval; HR, Hazard ratio.

**Figure 2:**
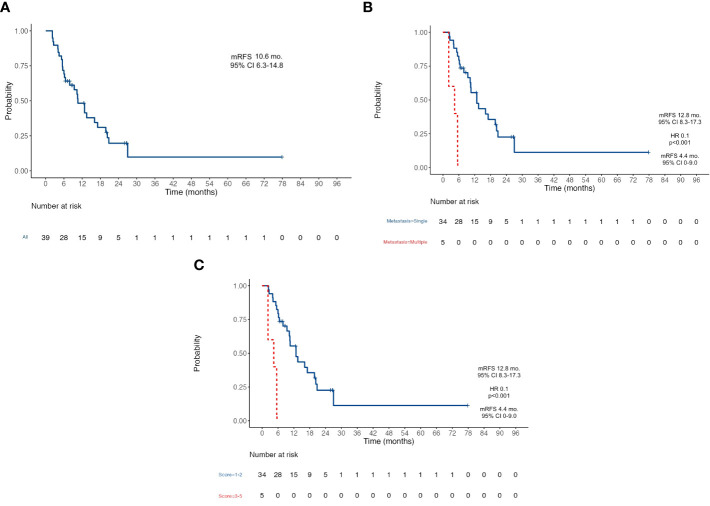
Kaplan-Meier survival curves for Recurrence-Free Survival RFS. **(A)** RFS in the whole population; **(B)** RFS according to the number of metastatic sites; **(C)** RFS according to the five-factors prognostic score. RFS, Recurrence-Free Survival; mFRS, Median Recur-rence-Free Survival; mo, Months; CI, Confidence Interval; HR Hazard ratio.

At the data cutoff analysis of November 2022, 17 patients were alive and, among them, 7 were free from any disease recurrence. Recurrence rate (RR) after surgery was 72%: in 78% of cases, disease relapse occurred in the peritoneum (22 cases); 11% (3) of patients progressed with liver metastases; one patient had a recurrence on both sites; one had only distant lymph node relapse; one patient progressed with multiple metastasis also involving brain and lungs. After a median follow-up of 33.6 months, median OS was 26.6 months (95% CI 23.8–29.4) (**Figure 1A**) and median RFS was 10.6 months (95% CI 6.3–14.8) (**Figure 2A**). Estimated 3-year OS was 13%. At univariate analysis, factors that significantly influenced OS were a good pathologic response according to the Mandard criteria [TRG 1–3, not reached (NR) versus 20.5 months for TRG 4–5, HR 0.23, *p* = 0.019] (**Figure 1B**), PS ECOG ≤ 1 (26.7 versus 11.2 months, HR 0.3, *p* = 0.022) (**Figure 1C**), and the number of metastatic sites: 26.7 months for patients with a single site versus 12.9 months for those with ≥2 sites (HR 0.34, *p* = 0.039) (**Figure 1D**). Having a single metastatic site was also the only variable that statistically significantly influenced RFS (12.8 vs. 4.4 months, HR 0.10, *p* < 0.001) (**Figure 2B**). The multivariate analysis confirmed having a single metastatic site as the only factor that statistically significantly influenced OS (HR 0.08, *p* = 0.048) but not RFS (**Supplementary Tables S2**, **S3**). Other clinicopathological features such as age, sex, histological type, HER-2 status, T-stage, N-stage, and primitive tumor site did not influence the benefits seen in these patients. In a study by Samarasam et al., patients were stratified according to four prognostic factors, demonstrating how the survival advantage of patients who underwent surgical resection disappeared when ≥3 negative factors were present (28). Thus, we tried to test an analogue score adding a relevant fifth prognostic factor: (1) pathological tumor growth (T+) on surgical specimen; (2) presence of liver metastases (L+); (3) peritoneum (P+) or (4) non-locoregional lymph nodes (N+); and (5) R2 resection. Moreover, in our population, there was a statistically significant difference between patients with a score of 1–2 and patients with a score of ≥3 in terms of both OS (26.7 vs. 12.9 months, HR 0.31, *p* = 0.023) (**Figure 1E**) and RFS (12.8 vs. 4.4 months, HR 0.10, *p* < 0.001) (**Figure 2C**).

## 4 Discussion

In recent years, OGC has become a recognized entity in clinical practice although a formal definition is still lacking (29). Despite chemotherapy treatment being the standard of care for advanced disease, increasing data from several series suggest that selected patients with limited metastatic spread might achieve long-term disease control and prolonged survival thanks to an aggressive multimodal strategy including surgery (11, 14, 28, 30–36). In the REGATTA trial, the authors explored the role of surgery for OGC with a single non-curable metastatic site, and results did not show any advantage for the surgical resection of the primary tumor (32). To note, up to 75% of the enrolled patients presented with peritoneal involvement such as non-curable metastasis, an independent poor prognostic factor (37). Instead, Markar et al. reported how combining metastasectomy and gastrectomy might be associated with improved survival without increasing postoperative mortality (34). Recently, the role of induction chemotherapy gained increasing attention, following early results from both clinical trials and real-world experience (12, 14, 31, 32, 38, 39). Han et al. found that patients with metastatic GC who were good responders to induction chemotherapy and who underwent curative R0 resection achieved an impressive median survival of 22.9 months (31). In our series, we collected real-world data from the repository of a high-volume tertiary referral institution (40). We strictly followed inclusion criteria comparable to the previous studies demonstrating a median OS extent to 26.6 months (95% CI 23.8–29.4) and a median RFS of 10.6 months (95% CI 6.3–14.8), overcoming any historical OS reported in first-line chemotherapy pivotal trials, which ranges approximately 9–11 months (9, 41, 42). In our study, how correct patient selection plays a key role clearly emerged, as revealed by the significant impact of the number of metastatic organs involved on OS (HR 0.34, *p* = 0.031) and RFS (HR 0.101, *p* < 0.001) rather than the specific site (L+, P+, or N+), provided that surgery was performed in a high-flow center after MTB discussion, as in our cases. Furthermore, the pivotal role of an experienced GC-dedicated MTB and the need for a repeated multidisciplinary evaluation have to be emphasized, not only at diagnosis but also during the treatment phase, owing to the fact that each patient deserves individual recommendations at any disease time point. Surgical radicality, defined as R0 resection, was achieved in 69% of patients, whereas in a minority of cases, a complete removal of all sites of metastasis was not technically feasible, mainly due to a greater extent of disease to the peritoneum than what we expected based on preoperative staging. Nevertheless, residual disease (R1–R2) seems not to affect OS as a single independent prognostic factor. In the same way, lymphadenectomy extent did not influence the benefit on OS. In each case, the best possible surgical procedure was performed in order to obtain the maximal cytoreduction and leave the patient free from macroscopical disease. In the majority of cases, a D2 lymphadenectomy was done, whereas a lymphadenectomy D3 was performed in a minority of patients based on the extent of disease in order to reach a radical resection as aforementioned. In contrast, patients who underwent only perigastric (D1) lymphadenectomy might be considered patients with a suboptimal surgical treatment, but their presence in the study, as well as the presence of R1–R2 resections, reflects a real-world surgical outcome and emphasizes how even these patients could obtain survival advantage from a combinatory strategy gaining a meaningful benefit from induction chemotherapy. Undoubtedly, the surgeon’s expertise significantly influenced the postoperative outcome of patients, revealing the gastrectomy plus lymphadenectomy combined with metastasectomy to be a safe procedure without significant improved morbidity. An emerging factor that also influenced OS was the efficacy of the preoperative antineoplastic systemic treatment; indeed, a good pathologic response such as Mandard TRG ≤ 3 was associated with better prognosis. In Oyama et al.’s retrospective study, comparing neoadjuvant versus adjuvant treatment in patients with GC and para-aortic lymph node metastasis who underwent surgical resection, 87.5% of patients in the first arm had a pathological response with 2-year OS and RFS rates of 93.8% and 75.0%, respectively (39). Despite the fact that pathological complete response (pCR) was not significantly associated with OS and RFS in the univariate analysis in our population, it is encouraging that patients who reached a pCR (TRG 1) on the surgical specimen had no evidence of disease relapse or death at data cutoff. These results identified whether surgery in a patient with OGC might be considered as another effective therapeutic line in a sequencing strategy. The major limitation of our study is its retrospective nature, which may entail selection bias and potential confounders. In our real-world experience, the indication to surgery was discussed in MTB for each patient at diagnosis. The indication to surgery was assessed again by our MTB after the completion of induction chemotherapy and on treatment radiological evaluation. Thus, we considered only patients who underwent surgery, not evaluating the rate of OGC that is potentially resectable at diagnosis and then becomes lost because of disease progression. Furthermore, the small sample size might have affected the results, limiting the power of our study and the statistical impact of some validated prognostic factors that appear not to have a significant role in survival outcome in our analyses. However, the small sample size reflects the reality of a monoinstitutional experience and the low rate of patients among the metastatic setting that might match the OGC definition criteria. Eventually, the final OS might have been influenced by chemotherapy protocols and number of cycles received during the neoadjuvant and adjuvant phases and all the subsequent systemic therapies administered on progressive disease. The variety in treatment regimens was an unavoidable bias as a consequence of the dramatic changes in the treatment landscape for patients with GC over the last 10 years, but due to this heterogeneity, it is not possible to estimate their impact on survival outcomes. In spite of these limits, our results send a clear message about the importance of a multimodal approach in patients with OGC and contribute to extend the evidence on current treatment options in this setting, which is still a matter of debate. The combined approach of induction chemotherapy followed by subsequent radical surgery on primitive tumor and synchronous metastases requires confirmation from large prospective randomized trials to build a new evidence-based standard of care. A first effort in this direction was the phase II FLOT-3 trial, which exploited the efficacy of induction chemotherapy followed by surgical resection in both limited and extensive metastatic GC with an overall median OS of 31.3 months for patients who underwent surgery, with a benefit of up to 1 year for those with limited metastatic spread compared to others (12). The ongoing phase III FLOT 5-RENAISSANCE trial further investigates this topic by enrolling patients with untreated OGC to be randomized 1:1 to undergo chemotherapy or surgical resection of primary tumor and metastases after induction chemotherapy (NCT02578368) (15). Likewise, the French SURGIGAST study compares the continuation of chemotherapy against a radical surgical approach in OGC (NCT03042169) (43).

## 5 Conclusions

Despite the limitations of the study due to its retrospective nature, it confirms the survival benefit of a sequential strategy of preoperative chemotherapy and radical surgical excision of both primary tumor and metastases in patients with OCG, providing a new treatment option for patients who are currently treated only with palliative chemotherapy.

## Data availability statement

The raw data supporting the conclusions of this article will be made available by the authors, without undue reservation.

## Ethics statement

The studies involving humans were approved by Institutional Ethics Committee of Fondazione Policlinico Agostino Gemelli IRCSS - Catholic University of Sacred Heart (protocol code 42028/19-ID2825). The study was conducted in accordance with the Declaration of Helsinki. The participants provided their written informed consent to participate in this study.

## Author contributions

MM: Conceptualization, Data curation, Formal analysis, Investigation, Methodology, Writing – original draft, Writing – review & editing. AV: Conceptualization, Data curation, Formal analysis, Investigation, Methodology, Validation, Writing – original draft, Writing – review & editing. MB: Conceptualization, Writing – review & editing. RV: Conceptualization, Writing – review & editing. ED: Conceptualization, Writing – review & editing. AB: Conceptualization, Writing – review & editing. AD: Conceptualization, Writing – review & editing. FR: Conceptualization, Writing – review & editing. VT: Conceptualization, Writing – review & editing. AA: Conceptualization, Writing – review & editing. GT: Conceptualization, Writing – review & editing. AS: Conceptualization, Writing – review & editing. CP: Conceptualization, Writing – review & editing.
