# Supplementary material for: Benefit of a multimodal approach combining chemotherapy and surgery in oligometastatic gastric cancer: experience from a tertiary referral center
Source: Front Oncol. 2024 Jun 7;14:1343596. doi: 10.3389/fonc.2024.1343596 (PMC11190071; doi:10.3389/fonc.2024.1343596)
Supplement: Supplementary file 1 [file Table_1.docx]

Supplementary Material

**Table S1**: Classification of post-operative complications according to Clavien-Dindo and Study Reported Incidence.

| **Grade** | **Definition** | **Patients’ number (%)** |
| --- | --- | --- |
| I | Any deviation from the normal postoperative course without the need for pharmacological treatment or surgical, endoscopic or radiological interventions (allowed therapeutic regimens are: drugs as antiemetics, antipyretics, analgesics, diuretics and electrolytes and physiotherapy). Wound infections opened at the bedside. | 5 (12.8) |
| II | Requiring pharmacological treatment with drugs other than such allowed for grade I complications (blood transfusions and total parenteral nutrition included). | 4 (10.2) |
| III  IIIa  IIIb | Requiring surgical, endoscopic or radiological intervention. |  |
|  | - Intervention not under general anesthesia  - Intervention under general anesthesia | 2 (5.1)  0 (0) |
| IV  IVa  IVb | Life-threatening complication (including CNS complications) requiring IC/ICU-management. |  |
|  | - single organ dysfunction (including dialysis)  - multi-organ dysfunction | 1 (2.5)  0 (0) |
| V | Death of a patient. | 0 (0) |

***CNS*** Central Nervous System complication (including brain hemorrhage, ischemic stroke, subarachnoidal bleeding, excluding transient ischemic attacks), ***IC*** Intermediate Care, ***ICU*** Intensive Care Unit.

**Table S2**: Univariate and Multivariate analysis for Overall Survival (OS).

|  |  |  | **UNIVARIATE** | | | **MULTIVARIATE** | | |
| --- | --- | --- | --- | --- | --- | --- | --- | --- |
| **Prognostic variables** | **n** | **Median OS (months)** | **HR** | **95% CI** | **p-value** | **HR** | **95% CI** | **p-value** |
| Gender  Male  Female |  |  |  |  |  |  |  |  |
|  | 16 | 28.8 | 0.88 | 0.38-2.06 | 0.771 | - | - | - |
|  | 23 | 26.6 |  |  |  |  |  |  |
| PS ECOG  0-1  2 |  |  |  |  |  |  |  |  |
|  | 33  6 | 26.7  11.2 | 0.30 | 0.11-0.84 | **0.022** | 0 | - | 0.924 |
| Primitive Tumor Location  Antrum/Pylorus  GEJ-Body/Fundus |  |  |  |  |  |  |  |  |
|  | 14  25 | 25.0  25.6 | 0.72 | 0.30-1.70 | 0.454 | - | - | - |
| Histotype  Intestinal  Diffuse/Mixed |  |  |  |  |  |  |  |  |
|  | 14  21 | 24.9  26.6 | 1.23 | 0.50-3.06 | 0.646 | - | - | - |
| Signet ring cell presence  No  Yes | 26  13 | 28.8  26.6 | 1.76 | 0.73-4.25 | 0.209 | - | - | - |
| HER-2 status  0-1+  2-3+ | 32  7 | 26.6  22.4 | 1.45 | 0.48-4.36 | 0.509 | - | - | - |
| Metastatic site  Liver only  Other sites | 6  33 | 28.8  26.6 | 0.95 | 0.32-2.85 | 0.923 | - | - | - |
| Metastatic site  Peritoneum only  Other sites | 22  17 | 26.7  24.9 | 0.65 | 0.28-1.51 | 0.320 | - | - | - |
| Metastatic site  Single  Multiple | 34  5 | 26.7  12.9 | 0.34 | 0.13-0.95 | **0.039** | 0.08 | 0.01-0.98 | **0.048** |
| Gastrectomy  Total  Subtotal | 23  16 | 26.6  25.0 | 0.95 | 0.40-2.22 | 0.898 | - | - | - |
| Lymphadenectomy  D1  D2-D3 | 5  34 | 24.9  26.6 | 0.67 | 0.19-2.32 | 0.526 | - | - | - |
| Residual disease  R0  R1-R2 | 27  12 | 26.6  24.9 | 1.26 | 0.54-2.97 | 0.591 | - | - | - |
| Mandard TRG  1-3  4-5 | 15  11 | Not Reached  20.5 | 0.23 | 0.69-0.79 | **0.019** | 0.31 | 0.07-1.44 | 0.134 |
| HIPEC  Yes  No | 19  20 | 23.9  26.7 | 1.18 | 0.51-2.72 | 0.710 | - | - | - |
| Score  1-2  3-5 | 34  5 | 26.7  12.9 | 0.31 | 0.11-0.85 | **0.023** | 0.07 | 0.01-1.22 | 0.068 |

***TRG*** Tumor Regression Grade, ***PS*** Performance Status, ***ECOG*** Eastern Cooperative Oncology Group, ***CI*** Confidence Interval.

**Table S3**: Univariate and Multivariate analysis for Recurrence-Free Survival (RFS).

|  |  |  | **UNIVARIATE** | | | **MULTIVARIATE** | | |
| --- | --- | --- | --- | --- | --- | --- | --- | --- |
| **Prognostic variables** | **n** | **Median RFS (months)** | **HR** | **95% CI** | **p-value** | **HR** | **95% CI** | **p-value** |
| Gender  Male  Female |  |  |  |  |  |  |  |  |
|  | 16 | 16.1 | 1.43 | 0.65-3.15 | 0.369 | - | - | - |
|  | 23 | 10.5 |  |  |  |  |  |  |
| PS ECOG  0-1  2 |  |  |  |  |  |  |  |  |
|  | 33  6 | 12.8  5.2 | 0.48 | 0.19-1.22 | 0.124 | - | - | - |
| Primitive Tumor Location  GEJ-Body/Fundus  Antrum/Pylorus |  |  |  |  |  |  |  |  |
|  | 25  14 | 10.5  10.6 | 0.93 | 0.44-1.97 | 0.848 | - | - | - |
| Histotype  Intestinal  Diffuse/Mixed |  |  |  |  |  |  |  |  |
|  | 14  21 | 12.8  10.3 | 0.70 | 0.31-1.58 | 0.384 | - | - | - |
| Signet ring cell presence  No  Yes | 26  13 | 12.8  6.2 | 1.72 | 0.80-3.70 | 0.166 | - | - | - |
| HER-2 status  0-1+  2-3+ | 32  7 | 12.8  10.5 | 0.96 | 0.33-2.82 | 0.944 | - | - | - |
| Metastatic site  Liver only  Other sites | 6  33 | 7.9  10.6 | 0.85 | 0.29-2.48 | 0.768 | - | - | - |
| Metastatic site  Peritoneum only  Other sites | 22  17 | 12.8  7.9 | 0.70 | 0.33-1.49 | 0.348 | - | - | - |
| Metastatic site  Single  Multiple | 34  5 | 12.8  4.4 | 0.10 | 0.03-0.34 | **<0.001** | 0.27 | 0.04-1.78 | 0.174 |
| Gastrectomy  Total  Subtotal | 23  16 | 10.5  12.8 | 0.71 | 0.33-1.53 | 0.382 | - | - | - |
| Lymphadenectomy  D1  D2-D3 | 5  34 | 5.2  12.8 | 0.40 | 0.14-1.24 | 0.115 | - | - | - |
| Residual disease  R0  R1-R2 | 27  12 | 12.8  10.5 | 0.97 | 0.44-2.13 | 0.942 | - | - | - |
| Mandard TRG  1-3  4-5 | 15  11 | 16.1  6.0 | 0.41 | 0.15-1.11 | 0.079 | - | - | - |
| HIPEC  Yes  No | 19  20 | 12.8  10.3 | 1.34 | 0.63-2.84 | 0.450 | - | - | - |
| Score  1-2  3-5 | 34  5 | 12.8  4.4 | 0.10 | 0.30-0.34 | **<0.001** | 0.27 | 0.04-1.80 | 0.174 |

***TRG*** Tumor Regression Grade, ***PS*** Performance Status, ***ECOG*** Eastern Cooperative Oncology Group, ***CI*** Confidence Interval
